# Association of heated tobacco product use and secondhand smoke exposure with suicidal ideation, suicide plans and suicide attempts among Korean adolescents: A 2019 national survey

**DOI:** 10.18332/tid/140824

**Published:** 2021-09-20

**Authors:** Soyoon Park, Kang-Sook Lee

**Affiliations:** 1 Department of Public Health, Graduate School, The Catholic University of Korea, Seoul, Korea; 2 Department of Preventive Medicine, College of Medicine, The Catholic University of Korea, Seoul, Korea

**Keywords:** heated tobacco product, SHS, smoking, suicide, adolescents

## Abstract

**INTRODUCTION:**

The consumption of heated tobacco products (HTPs) is increasing among adolescents worldwide. Although suicide and HTP use are linked, the association between suicide-related behavior, HTP use, and indirect smoking exposure are not yet properly studied. This study examined the association of HTP use and exposure to secondhand smoke (SHS) with suicidal ideation, suicide plans, and suicide attempts among South Korean adolescents.

**METHODS:**

Data from 57303 respondents (95.3% response rate) were obtained from the 2019 Korean Youth Risk Behavior Web-based Survey. Chi-squared tests and multivariable logistic regression analyses were used to examine the association of HTP use and SHS exposure with suicidal ideation, suicide plans, and suicide attempts among adolescents. Multivariable logistic regression analyses included: Model 1, which was adjusted for demographic characteristics such as sex, school type, perceived school performance, economic status, and residence type; and Model 2, which was adjusted for demographics, depression, and drug use.

**RESULTS:**

The risk of suicidal ideation was 1.37 (95% CI: 1.10–1.70) and 1.44 (95% CI: 1.18–1.75) times higher among HTP users who were exposed to SHS at home and at public places, respectively, compared to non-users. The risk of suicide attempts was 1.88 (95% CI: 1.37–2.57), 1.45 (95% CI: 1.63–2.00), and 2.21 (95% CI: 1.63–3.00) times higher among HTP users exposed to SHS at home, school, and at public places, respectively.

**CONCLUSIONS:**

HTP use, and SHS exposure are likely indicators of risk behaviors. Our findings suggest possible directions for initiating, implementing, and evaluating programs and services to monitor HTP use and SHS exposure among Korean adolescents.

## INTRODUCTION

Suicide is a major mental health issue among adolescents worldwide^1^. Adolescent suicide is an alarming reality in South Korea, as well as globally. The suicide rate among Korean adolescents is twice the average rate reported in the Organization for Economic Cooperation and Development data, which makes it the highest among the 37 member countries. The suicide rate of Korean adolescents has progressively increased over the years, with a 17.8% increase from 2017 to a death rate of 9.1 per 100000 in 2018^2^. Korean adolescents (in 2019) faced suicide thoughts (13.1%), had suicide plans (4%), and suicide attempts (3%)^3^. According to one study, 33.4% of people who think about suicide planned suicide, and 33.9% of those who think about suicide try to commit suicide^4^.

Several studies have reported an association between smoking and suicidal behaviors in adolescents^5-7^ and conventional and e-cigarette consumption and exposure to SHS have been found to be significantly associated with adolescent suicidality^5,8^.

In Korea, ever use and current use of e-cigarettes among adolescents increased from 7.4 to 7.9% and from 2.2 to 2.7%, respectively, between 2017 and 2018^9^. Results of recent research indicate that awareness, experience, and current use of heated tobacco products (HTPs) have rapidly increased among Korean young adults, with respondents believing that HTPs would help them quit smoking and are less harmful to their health than conventional cigarettes^10^.

E-cigarettes and HTPs are similar in certain aspects, such as the resemblance of the device and process. However, they are essentially different. E-cigarettes are battery-powered devices that deliver nicotine to the user through vaporization of a propylene-glycol solution, typically in combination with flavors^11^. In contrast, HTPs are defined as ‘tobacco products that produce aerosols containing nicotine and other chemicals that are inhaled by users through the mouth’^12^.

HTPs were introduced in Korea in June 2017, and the market share currently accounts for 10.8% of the total national tobacco market^13^. In the 2018 Korea Youth Risk Behavior Web-based Survey (KYRBWS), 81.3% of Korean youth respondents were smokers comprising traditional cigarettes, electronic cigarettes, and HTP users^14^.

A meta-analysis indicated an association between smoking and suicide possibly due to the biological pathway of smoking itself or relationship with other high-risk behaviors such as alcohol and drug dependency^6^. Furthermore, several studies have shown a significant association between depression and smoking^15,16^. Suicide is higher when one experiences pain and despair, and in this state, tobacco and drug use have been associated with increased suicide attempts^17^. Individuals experiencing suicidality tend to develop the ability to overcome the fear of death^18,19^. In a study of people aged 13–18 years who visited the emergency room in a suicide attempt, tobacco use increased^20,21^. Moreover, secondhand smoke (SHS) exposure location is significantly associated with depressive symptoms in former and current smokers^22^. In establishing a policy to prevent suicide of adolescents, active interventions of risk factors related to depression and smoking are needed^23^.

In addition, another perspective that strengthens an association between cigarette use and suicidality stems from the generation of toxic constituents during cigarette use. Smoking reduces serotonin and monoamine oxidase levels, which can cause severe depression and suicide in some smokers^24^. HTPs release aerosols containing nicotine and toxic chemicals upon heating of tobacco without combustion^25^. Despite sufficient research that links tobacco use to suicide, the association between HTP use and suicide-related behaviors among adolescents has not been investigated. There is limited research on the association between HTP use, exposure to toxic constituents, and suicidality. A recent study found that HTPs might be used as a tool for suicide attempts^26^.

SHS requires attention in suicide research because studies have discovered that SHS exposure could be associated with suicide attempts, albeit weakly^5^, particularly with heavy exposure (>20 cigarettes/ day) in some adolescents^20^. Exposure to SHS among children and adolescents increases their serum cotinine levels, which can induce anxiety and mental disorders^27^. However, the association of SHS experiences with suicidality in Korean adolescents has not been investigated.

This study aimed to examine the association between HTP use and suicidal ideation, suicide plans, and suicide attempts among Korean adolescents. In addition, this study investigated whether SHS exposure in different circumstances (e.g. home, school, and public places) and its interactions with HTP use are associated with suicidality.

## METHODS

### Study population

Data were obtained from the 2019 KYRBWS, which has been conducted annually by the Korea Centers for Disease Control and Prevention (KCDC) since 2005. The 2019 KYRBWS was conducted in April 2019^10^. The survey data are publicly available. All data analyses were conducted in accordance with KCDC guidelines. To obtain a nationally representative sample, the KYRBWS was administered with a stratified three-stage random cluster sampling method. In the first stage, the study population was stratified according to the geographical region and school type to minimize any sampling error. In the second stage, 400 middle schools and 400 high schools were selected by proportional sampling to match the study population. In the third stage, the sample schools were sorted out using systematic sampling, and sample classes were chosen through simple randomization sampling of the selected schools. This web-based survey comprised 105 questions for 15 health-related areas, including cigarette use behavior and mental health. Participants did not fill in any personal information. This study was approved by the Institutional Review Board of the C University of Korea (MC20ZESI0102).

### Measurements

#### Sociodemographic factors

Sociodemographic factors included the participants’ sex, school type, self-perceived academic achievement in the past year, perceived economic status, and residence type. School types consisted of middle school and high school. Academic achievements and economic status included low, middle low, middle, middle high, and high categories. Residence types comprised that of immediate family, relatives, boarding house, dormitory, and social welfare care (Table 1).

**Table 1:** Participants’ general characteristics, South Korea, 2019 (N=57303)

*Variable*	*All (n=57303) n (%)*	*Male (n=29841) n (%)*	*Female (n=27462) n (%)*	p*
**School type**				
Middle	29384 (51.3)	15401 (51.6)	13983 (50.9)	0.577
High	27919 (48.7)	14440 (48.4)	13479 (49.1)	
**Perceived academic achievement**				
High	7647 (13.3)	4661 (15.6)	2986 (10.9)	<0.001
Middle high	14296 (25.0)	7254 (24.3)	7042 (25.6)	
Middle	17234 (30.1)	8615 (28.9)	8619 (31.4)	
Middle low	12570 (21.9)	6251 (20.9)	6319 (23.0)	
Low	5556 (9.7)	3060 (10.3)	2496 (9.1)	
**Perceived economic status**				
High	6379 (11.1)	4019 (13.5)	2360 (8.6)	<0.001
Middle high	16126 (28.2)	8566 (28.7)	7560 (27.5)	
Middle	27457 (47.9)	13561 (45.4)	13896 (50.6)	
Middle low	6042 (10.5)	2950 (9.9)	3092 (11.3)	
Low	1299 (2.3)	745 (2.5)	554 (2.0)	
**Residence type**				
With immediate family	54267 (94.7)	28177 (94.4)	26090 (95.0)	<0.001
With relatives	332 (0.6)	197 (0.7)	135 (0.5)	
Boarding house	347 (0.6)	215 (0.7)	132 (0.5)	
Dormitory	2126 (3.7)	1111 (3.7)	1015 (3.7)	
Social welfare care setting	231 (0.4)	141 (0.5)	90 (0.3)	
**Depression**				
No	41275 (72.0)	23346 (78.2)	17929 (65.3)	<0.001
Yes	16028 (28.0)	6495 (21.8)	9533 (34.7)	
**Drug use**				
No	56700 (98.9)	29515 (98.9)	27185 (99.0)	0.326
Yes	603 (1.1)	326 (1.1)	277 (1.0)	

* p<0.001 indicates significant differences between the male and female groups in the results of the chi-squared tests.

#### Lifetime and current cigarette use

Study respondents were asked about their lifetime experience of conventional-cigarette-only use, e-cigarette use, and HTP-only use as a binary result (yes/no). Responses were measured with the questions: ‘Have you ever taken even one puff of a cigarette in your lifetime?’, ‘Have you ever used e-cigarettes in your lifetime?’, and ‘Have you ever used HTPs in your lifetime?’. Only those who answered ‘Yes’ to lifetime cigarette use responded to current cigarette use. Participants were assessed for their current use of HTPs, conventional cigarettes, and e-cigarettes by asking: ‘Have you used cigarettes in the past 30 days?’, ‘Have you used e-cigarettes in the past 30 days?’, and ‘Have you used HTPs in the past 30 days?’.

#### SHS

To assess SHS exposure, participants were asked: ‘Have you been exposed to SHS at home in the past 7 days?’, ‘Have you been exposed to SHS at school in the past 7 days?’, and ‘Have you experienced SHS in public in the past 7 days?’.

#### Depression

This study included depression as a confounding variable in the experimental model because depression is known to be a significant factor for suicidality. The inclusion of this variable could reveal the true association of cigarette use with suicidality in this study by excluding the predictability of depression. Depression was assessed based on the question: ‘Have you felt too sad or desperate to have a daily life for 2 weeks straight during the past 12 months?’. Binary response options of ‘no’ or ‘yes’ were used.

#### Drug use

Have you ever habitually or deliberately taken drugs, ‘drank’ butane gas, or ‘bond’? Binary response options of ‘no’ or ‘yes’ were used.

#### Suicidality

Suicide-related perceptions and behaviors were measured with three questions about suicidal ideation, suicide plans, and suicide attempts as follows: ‘Have you thought seriously about committing suicide in the past 12 months?’, ‘Have you made specific plans to commit suicide in the past 12 months?’, and ‘Have you attempted suicide in the past 12 months?’. Binary response options of no or yes were used.

### Statistical analyses

The general characteristics of the study population and suicidality according to lifetime cigarette use, current cigarette use, and SHS experiences, were analyzed using chi-squared tests. Chi-squared tests were also used to determine significant differences among demographic characteristics, cigarette use, and SHS in sex-stratified analyses. Concerning the association between cigarette use and suicidality, chi-squared tests were used to ascertain significant differences by cigarette use and non-use.

To assess the association of current and lifetime use patterns with suicidal ideation, suicide plans, and suicide attempts, a multivariable logistic regression analysis was performed after adjusting for confounding variables (i.e. sex, school type, perceived school performance, economic status, residence type, and depression). Significance was set at p<0.05. In the first test, demographic confounding variables were added. In the second test, demographics, depression, and drug use were added as confounders. The confounding variables were entered initially, and the independent variables were entered in the second regression model. The interaction effects between HTP use and SHS exposure for suicidality were tested by multiplying the lifetime HTP use and SHS exposure to predict suicidality. According to the variance inflation factor values, every variable had a value lower than 10; therefore, there was no collinearity. All statistical analyses were conducted using SPSS 25.0 (IBM, Armonk, NY, USA, 2017).

## RESULTS

### Participants’ general characteristics

Table 1 presents participants’ demographic characteristics. Of the 57303 participants, 51.3% were middle school students, and 48.7% were high school students. Most (94.7%) lived with their immediate families, 0.6% lived with relatives, 0.6% lived in boarding houses, 3.7% lived in dormitories, and 0.4% lived in social welfare care settings. Further, 28% experienced depression and 1.1% used drugs.

Table 2 presents the smoking-related characteristics of the participants. Specifically, 11.3% of the participants had consumed cigarettes for lifetime use, and 52.2% used cigarettes currently. Moreover, 42.6% were currently using e-cigarettes, and 52.2% (51.6% boys and 54.4% girls) were current HTP users. The SHS exposure locations were house, school, and public places in 32.2%, 21%, and 51.7% of the cases, respectively. The rates of suicide plans and attempts were 4% and 3%, respectively. Female respondents were almost twice as likely as male respondents to have attempted suicide.

**Table 2:** Smoking-related characteristics of the participants, South Korea, 2019 (N=57303)

*Variable*	*All (n=57303) n (%)*	*Male (n=29841) n (%)*	*Female (n=27462) n (%)*	p*
**Conventional cigarette**				
**Lifetime use**				
No	50227 (87.7)	24842 (83.2)	25385 (92.4)	<0.001
Yes	7076 (11.3)	4999 (16.8)	2077 (7.6)	
**Current use**				
No	3383 (47.8)	2369 (47.4)	1014 (48.8)	0.273
Yes	3693 (52.2)	2630 (52.6)	1063 (51.2)	
**E-cigarette**				
**Lifetime use**				
No	53268 (93.0)	26673 (89.4)	26595 (96.8)	<0.001
Yes	4035 (7.0)	3168 (10.6)	867 (3.2)	
**Current use**				
No	2317 (57.4)	1861 (58.7)	456 (52.6)	<0.001
Yes	1718 (42.6)	1307 (41.3)	411 (47.4)	
**HTP**				
**Lifetime use**				
No	54630 (95.3)	27771 (93.1)	26859 (97.8)	<0.001
Yes	2673 (4.7)	2070 (6.9)	603 (2.2)	
**Current use**				
No	1277 (47.8)	1002 (48.4)	275 (45.6)	0.226
Yes	1396 (52.2)	1068 (51.6)	328 (54.4)	
**SHS**				
**Home**				
No	38858 (67.8)	20998 (70.4)	17860 (65.0)	<0.001
Yes	18445 (32.2)	8843 (29.6)	9602 (35.0)	
**School**				
No	45270 (79.0)	23860 (80.0)	21410 (78.0)	<0.001
Yes	12033 (21.0)	5981 (20.0)	6052 (22.0)	
**Public places**				
No	27655 (48.3)	16257 (54.5)	11398 (41.5)	<0.001
Yes	29648 (51.7)	13584 (45.5)	16064 (58.5)	
**Suicidality**				
**Ideation**				<0.001
No	49805 (86.9)	27110 (90.8)	22695 (82.6)	
Yes	7498 (13.1)	2731 (9.2)	4767 (17.4)	
**Plan**				<0.001
No	54997 (96.0)	28937 (97.0)	26060 (94.9)	
Yes	2306 (4.0)	904 (3.0)	1402 (5.1)	
**Attempt**				<0.001
No	55572 (97.0)	29275 (98.1)	26297 (95.8)	
Yes	1731 (3.0)	566 (1.9)	1165 (4.2)	

* p<0.001 indicates significant differences between the male and female groups in the results of the chi-squared tests.

### Suicidality by cigarette use pattern

Table 3 presents the prevalence of suicidality by cigarette-use pattern. The rates of suicide ideation, planning, and attempts for people who never used tobacco in their lifetime were 11.9%, 3.4%, and 2.4%, respectively; however, among those who were lifetime tobacco users, the proportions were 21.3%, 8.2%, and 7.2%, respectively (p<0.001). Among the current e-cigarette users, the rates of suicidal ideation, planning, and attempts were 26.7%, 12.9%, and 12.4%, respectively, which were significantly different from the proportions of 16.2%, 6.3%, and 4.8%, respectively, among non-users (p<0.001). In addition, 26.8%, 13.9%, and 13.8% of the current HTP users experienced suicide thoughts, plans, and attempts, respectively, and the suicidal relevance was the highest among the types of cigarettes (p<0.001). Suicide ideation, planning, and attempt rate were significantly associated with the place of exposure to SHS (16.7%, 5.5%, and 4.3%, respectively), exposure at school (17.6%, 6.3%, and 4.9%, respectively), and exposure at public places (15.9%, 4.9%, and 3.8%, respectively; p<0.001). The rates of suicide ideation, plan, and attempts were 16.7%, 5.5%, and 4.3%, respectively, at home, and 17.6%, 6.3%, and 4.9%, respectively, at school as the SHS location; these were significantly different from the rates observed when home and school were not the SHS locations (all p<0.001).

**Table 3:** Prevalence of suicidality by cigarette-use pattern among Korean adolescents, South Korea, 2019 (N=57303)

*Variable*	*Suicidal ideation n (%)*		p*	*Suicide plan n (%)*		p*	*Suicide attempt n (%)*		p*
	*No*	*Yes*		*No*	*Yes*		*No*	*Yes*	
**Conventional cigarette**									
**Lifetime use**									
No	44234 (88.1)	5993 (11.9)	<0.001	48504 (96.6)	1723 (3.4)	<0.001	49004 (97.6)	1223 (2.4)	<0.001
Yes	5571 (78.7)	1505 (21.3)		6493 (91.8)	583 (8.2)		6568 (92.8)	508 (7.2)	
**Current use**									
No	2765 (81.7)	618 (18.3)	<0.001	3172 (93.8)	211 (6.2)	<0.001	3226 (95.4)	157 (4.6)	<0.001
Yes	2806 (76.0)	887 (24.0)		3321 (89.9)	372 (10.1)		3342 (90.5)	351 (9.5)	
**E-cigarette**									
**Lifetime use**									
No	44604 (87.5)	6664 (12.5)	<0.001	51331 (96.4)	1937 (3.6)	<0.001	51862 (97.4)	1406 (2.6)	<0.001
Yes	3201 (79.3)	834 (20.7)		3666 (90.9)	369 (9.1)		3710 (91.9)	325 (8.1)	
**Current use**									
No	1942 (83.8)	375 (16.2)	<0.001	2170 (93.7)	147 (6.3)	<0.001	2205 (95.2)	112 (4.8)	<0.001
Yes	1259 (73.3)	459 (26.7)		1496 (87.1)	222 (12.9)		1505 (87.6)	213 (12.4)	
**HTP**									
**Lifetime use**									
No	44753 (87.4)	6877 (12.6)	<0.001	52625 (96.3)	2005 (3.7)	<0.001	53175 (97.3)	1455 (2.7)	<0.001
Yes	2052 (76.8)	621 (23.2)		2372 (88.7)	301 (11.3)		2397 (89.7)	276 (10.3)	
**Current use**									
No	1030 (80.7)	247 (19.3)	<0.001	1170 (91.6)	107 (8.4)	<0.001	1193 (93.4)	84 (6.6)	<0.001
Yes	1022 (73.2)	374 (26.8)		1201 (86.1)	194 (13.9)		2397 (89.7)	192 (13.8)	
**SHS**									
**Home**									
No	34432 (88.6)	4426 (11.4)	<0.001	37566 (96.7)	1292 (3.3)	<0.001	37927 (97.6)	931 (2.4)	<0.001
Yes	15373 (83.3)	3072 (16.7)		17431 (94.5)	1014 (5.5)		17645 (95.7)	800 (4.3)	
**School**									
No	39890 (88.1)	5380 (11.9)	<0.001	43717 (96.6)	1553 (3.4)	<0.001	44130 (97.5)	1140 (2.5)	<0.001
Yes	9915 (85.4)	2118 (17.6)		11280 (93.7)	753 (6.3)		11441 (95.1)	591 (4.9)	
**Public places**									
No	24865 (89.9)	2790 (10.1)	<0.001	26788 (96.9)	867 (3.1)	<0.001	27064 (97.9)	591 (2.1)	<0.001
Yes	24940 (84.1)	4708 (15.9)		28209 (95.1)	1439 (4.9)		28508 (96.2)	1140 (3.8)	

* p<0.001 indicates significant differences between the user and non-user groups in the results of the chi-squared tests.

Association of suicidality with cigarette-use pattern Table 4 shows the results of the multivariable logistic regression analysis regarding suicidality according to the type of tobacco use. Model 1 was adjusted for sex, school type, perceived school performance, economic status, and residence type. In Model 1, the lifetime users of HTPs were 2.4 times more likely to report suicide attempts (AOR=2.40; 95% CI: 1.92–2.97, p<0.001) than non-smokers, and recent HTP users were 1.7 times more likely to report suicide attempts (AOR=1.70; 95% CI: 1.08–2.67, p<0.05). Participants who experienced SHS at home demonstrated significantly higher suicidal ideation (AOR=1.29; 95% CI: 1.22–1.36, p<0.001), planning (AOR=1.38; 95% CI: 1.27–1.51, p<0.001), and attempts (AOR=1.43; 95% CI: 1.29–1.58, p<0.001) than those who were not exposed to SHS at home. Among HTP users who were exposed to SHS at home, school, and at public places, the likelihood of suicide attempts was 2.57 (95% CI: 1.91–3.47, p<0.001), 1.94 (95% CI: 1.44–2.60, p<0.001), and 2.76 (95% CI: 2.04–3.71, p<0.001) times higher, respectively.

**Table 4:** Association of cigarette use with suicidality, South Korea, 2019 (N=57303)

*Variable*	*Non-use (Ref.)*	*Suicidal ideation AOR (95% CI)*	*p*	*Suicide plan AOR (95% CI)*	*p*	*Suicide attempts AOR (95% CI)*	*p*
**Model 1^a^**							
**Lifetime use**							
Conventional cigarette	1	2.10 (1.92–2.29)	<0.001	2.17 (1.89–2.50)	<0.001	2.71 (2.32–3.17)	<0.001
E-cigarette	1	1.08 (0.95–1.23)	0.242	1.25 (1.03–1.52)	<0.050	1.31 (1.05–1.63)	<0.050
HTP	1	1.43 (1.25–1.64)	<0.001	1.99 (1.63–2.43)	<0.001	2.40 (1.92–2.97)	<0.001
**Current use**							
Conventional cigarette	1	0.91 (0.66–1.26)	0.572	0.95 (0.59–1.53)	0.841	0.85 (0.51–1.41)	0.527
E-cigarette	1	1.73 (1.30–2.31)	<0.001	2.06 (1.34–3.15)	<0.010	2.26 (1.44–3.57)	<0.001
HTP	1	1.25 (0.93–1.68)	0.134	1.29 (0.85–1.96)	0.232	1.70 (1.08–2.67)	<0.050
**SHS**							
Home	1	1.29 (1.22–1.36)	<0.001	1.38 (1.27–1.51)	<0.001	1.43 (1.29–1.58)	<0.001
School	1	1.39 (1.31–1.48)	<0.001	1.70 (1.55–1.87)	<0.001	1.78 (1.60–1.87)	<0.001
Public places	1	1.38 (1.31–1.45)	<0.001	1.24 (1.13–1.36)	<0.001	1.38 (1.24–1.53)	<0.001
**HTP + SHS**							
HTP + SHS home	1	1.74 (1.43–2.13)	<0.001	2.07 (1.57–2.73)	<0.001	2.57 (1.91–3.47)	<0.001
HTP + SHS school	1	1.46 (1.18–1.79)	<0.001	2.06 (1.55–2.73)	<0.001	1.94 (1.44–2.60)	<0.001
HTP + SHS public places	1	1.78 (1.48–2.14)	<0.001	2.04 (1.55–2.68)	<0.001	2.76 (2.04–3.71)	<0.001
**Model 2^b^**							
**Lifetime use**							
Conventional cigarette	1	1.64 (1.49–1.80)	<0.001	1.63 (1.42–1.88)	<0.001	2.00 (1.71–2.34)	<0.001
E-cigarette	1	0.99 (0.86–1.14)	0.866	1.10 (0.90–1.35)	0.368	1.16 (0.93–1.46)	0.194
HTP	1	1.20 (1.03–1.40)	<0.050	1.56 (1.26–1.93)	<0.001	1.90 (1.51–2.39)	<0.001
**Current use**							
Conventional cigarette	1	0.98 (0.93–1.04)	0.480	1.00 (0.92–1.08)	0.961	1.02 (0.94–1.10)	0.683
E-cigarette	1	1.12 (1.04–1.21)	<0.010	1.19 (1.08–1.32)	<0.010	1.19 (1.07–1.32)	<0.010
HTP	1	1.04 (0.96–1.12)	0.372	1.06 (0.95–1.17)	0.314	1.05 (0.95–1.17)	0.349
**SHS**							
Home	1	1.19 (1.12–1.26)	<0.001	1.22 (1.12–1.34)	<0.001	1.25 (1.13–1.39)	<0.001
School	1	1.18 (1.11–1.26)	<0.001	1.39 (1.29–1.53)	<0.001	1.43 (1.27–1.60)	<0.001
Public places	1	1.20 (1.13–1.27)	<0.001	1.05 (0.96–1.16)	0.314	1.16 (1.04–1.30)	<0.010
**HTP + SHS**							
HTP + SHS home	1	1.37 (1.10–1.70)	<0.010	1.45 (1.09–1.95)	<0.050	1.88 (1.37–2.57)	<0.001
HTP + SHS school	1	1.21 (0.96–1.52)	0.108	1.57 (1.16–2.12)	<0.010	1.45 (1.06–2.00)	<0.050
HTP + SHS public places	1	1.44 (1.18–1.75)	<0.001	1.64 (1.23–2.17)	<0.001	2.21 (1.63–3.00)	<0.001

a Model 1 was adjusted for sex, school type, perceived school performance, economic status, and residence type. Model fit statistics were: χ^2^=194.495–656.331, df=3, p<0.001, Nagelkerke R^2^=0.055–0.135 for suicidal ideation; χ^2^=31.512–486.223, df=3, p<0.001 and Nagelkerke R^2^=0.042–0.176 for suicide plan; and χ^2^=48.631–633.291, df=3, p<0.001, Nagelkerke R^2^=0.065–0.206 for suicide attempts.

b Model 2 was adjusted for depression, and drug use, along with all factors that were adjusted in Model 1. Model fit statistics were: χ^2^=476.314–8811.696, df=10, p<0.001, Nagelkerke R^2^=0.261–309 for suicidal ideation; χ^2^=367.766–3268.573, df=10, p<0.001, Nagelkerke R^2^=0.188–0.327 for suicide plan; χ^2^=361.876–3076.672, df=10, p<0.001, Nagelkerke R^2^=0.206–0.331 for suicide attempts.

Model 2 controlled for depression and drug use along with all the factors controlled in Model 1. For the use of tobacco adjusted for demographic, depression, and drug use in Model 2, the results were similar to those of Model 1, except for a few associations. Lifetime users of HTPs were 1.9 times more likely to report suicide attempts than the non-users (95% CI: 1.51–2.39, p<0.001). Participants were exposed to SHS at home and school, the risks of suicide attempts were 1.25 (95% CI: 1.13–1.39, p<0.001) and 1.43 (95% CI: 1.27–1.60, p<0.001) times higher, respectively, than those who were not exposed. When HTP users were exposed to SHS at home, the risks of suicidal ideation (AOR=1.37; 95% CI: 1.10–1.70, p<0.01) and suicide plan (AOR=1.45; 95% CI: 1.09–1.95, p<0.05) were significant. Similarly, when HTP users were also exposed to SHS at public places, the likelihood of suicide ideation (AOR=1.44; 95% CI: 1.18–1.75, p<0.001) and suicide plans (AOR=1.64; 95% CI: 1.23–2.17, p<0.001) were significantly higher compared to HTP users who were not exposed to SHS. When HTP users were exposed to SHS, the likelihood of suicide attempt was 1.88 (95% CI: 1.37–2.57, p<0.001) times higher when exposure was at home, 1.45 (95% CI: 1.63–2.00, p<0.05) times higher at school, and 2.21 (95% CI: 1.63–3.00, p<0.001) times higher at public places.

## DISCUSSION

The nationwide youth survey found that adolescents using HTPs at least once in their lives had a higher likelihood of suicidal thoughts, suicide plans, and suicide attempts. When HTP users were also exposed to SHS at home and at public places, the likelihood of suicidal ideation was 1.37 and 1.44 times higher, respectively. When HTP users were exposed to SHS, the likelihood of reported suicide attempts increased by 1.88 times at home, 1.45 times at school, and 2.21 times at public places.

According to a study on the relationship between e-cigarette use and suicide in adolescents, suicidal ideation, suicide plans, and serious suicide attempts increased by 1.58, 2.44, 3.09 times, respectively, in the adolescents who used e-cigarettes compared to those who did not use e-cigarettes in the past 30 days^28^. In another study, smoking was a predominant factor affecting suicide thoughts, plans, and attempts of adolescents; the number of cigarettes smoked was related to suicide^29^. The current study demonstrated that smoking was an important factor that affected suicidal ideation, plans, and attempts among adolescents. Furthermore, the amount of smoking was related to suicide, making our findings consistent with recent studies.

In a meta-analysis of prospective cohort studies for evaluation of cigarette smoking and risk of completed suicide, current smokers had a 1.81-fold higher risk of suicide than non-smokers, and a 24 percent increase in the risk of suicide was observed with each increment of 10 cigarettes per day^30^. Similar to the results of previous studies, in our study, the likelihood of reporting suicide attempts was 2.71 times higher in lifetime tobacco users and 2.4 times higher in lifetime HTP users than non-smokers. Furthermore, these results showed that the suicide attempt rates were 2.0 and 1.9 times higher among lifetime tobacco and HTP users, respectively, even after controlling for depression and drug use as additional confounding variables. However, the AORs for suicide attempts in lifetime e-cigarette users were not significant. In our study, indirect smoking at home, schools, and public places was associated with suicide, which was significant even after adjusting for depression and drug use. The likelihood of suicidal ideation, plans, and attempts were higher for those who used HTPs in addition to exposure to SHS than those who were exposed to SHS only.

HTP users perceive that HTPs are less harmful than traditional cigarettes, however further research is needed as HTPs contain a higher level of tar than conventional cigarettes, which contradicts this assumption^31^. Recently, the use of HTPs in Koreans has increased continuously, with 90% of HTP users consuming e-cigarettes or conventional cigarettes, simultaneously. Currently, HTP users in Korea are likely to be multiple users^32^. Furthermore, smokers also use e-cigarettes and HTPs together. However, in a 2014 meta-analysis, the rate of success in smoking cessation fell to 39 per cent^33^. Another meta-analysis conducted in 2016 showed that e-cigarette users had a 28% lower success in stopping smoking than people who did not use e-cigarettes^29^.

In a study of New Zealanders aged 15–21 years, people looking for new tobacco products, such as e-cigarettes and HTPs, increase their suicidal behavior in adolescence, even controlling for variables such as mental health and depression^34^. Adolescence is a period when the development of the brain circuit that regulates cognition and emotion is vulnerable to the effects of nicotine and tobacco use. Exposure to harmful ingredients can cause anxiety and depression in adolescents^20,21,35^. The causal relationship between nicotine and suicide is unclear, but nicotine exposure disrupts the brain dopamine pathway, increasing stress and depressive symptoms^36^.

Addiction to nicotine can lead to awareness of HTPs^10^ and behavior related to suicide, and tobacco use increases the risk of suicide. Moreover, it has been reported that smoking initiation at a younger age increased nicotine dependence and negatively affected mental health^37^. Smokers have been found to have lower cerebrospinal fluid levels of serotonin metabolites. In a study, smokers showed a 2.6-fold higher suicide attempt risk, lower cerebrospinal fluid levels of serotonin metabolites, and lower platelet monoamine oxidases^38^. Lower serotonin levels could cause depression and increase aggressive and impulsive behavior depending on their existing characteristics and temperaments.

Another probable factor regarding HTP use in Korea might be adolescents’ accessibility to cigarettes. The Korean Youth Protection Act banned the access of cigarettes for adolescents, however, a recent survey found that 69.9% and 66.4% of male and female adolescents, respectively, who attempted to purchase cigarettes were able to obtain them at convenience stores^9^. The availability of HTPs since 2017 and closed system e-cigarette vapor products (e.g. JUUL) since 2019 in Korea has transformed Korea’s tobacco market, capturing 11.8% of the market share in 2019^39^; this suggests that regulations on adolescent access to e-cigarettes are still lacking. Therefore, providing Korean adolescents with proper interventional and preventive programs for both HTP use and mental disorders may facilitate better identification of risk symptoms, including suicide-related perceptions and behaviors.

### Limitations

This study has some limitations. First, this cross-sectional study design precluded the detection of any causal associations between HTP use and suicidality and hence only associations can be assessed. Future research should include a longitudinal panel analysis to examine the influence of cigarette-use behaviors on subsequent suicidality. Second, the respondents might have overrated socially desirable responses (e.g. school grades) and underrated negative responses (e.g. cigarette use and suicidality), which may have caused response biases. Third, the use of cigarettes and HTPs was assessed based on a single item, which asked participants to choose a binary response between ever or never use. The single item would neither fully represent the respondents’ cigarette-use habits nor was it derived from scales that were validated in earlier studies. Therefore, the measures of cigarette use, SHS exposure, suicidal ideation, suicide plans, and suicide attempts need to be further validated in future research. Fourth, multifaceted aspects of HTP use and risk behaviors predicting suicidal ideation, suicide plans, and suicide attempts were not investigated in this study. Cigarette use, substance use, and other psychological factors could possibly explain suicidality^40^. Fifth, additional mental disorder factors, such as psychosis and loneliness, could be included in the analysis as confounders. With the inclusion of the relevant variables, the true associations between HTP use, SHS exposure, and suicidality can be verified.

## CONCLUSIONS

This study revealed the possibility that Korean adolescents with HTP use and SHS exposure may have other risk symptoms that are associated with and predict suicidal ideation, suicide plans, and suicide attempts. The results call for an evaluation and intervention amid the expansion of HTP use in the Korean adolescent population. Policymakers and healthcare professionals should initiate cigarette-use cessation programs that include HTP use and SHS exposure for adolescents. Through screening efforts, adolescents with HTP use, SHS exposure, mental health risk factors, and suicidality could receive targeted interventions. The government can refine policies on adolescents’ access to tobacco products, including HTPs, e-cigarettes, and conventional cigarettes. National campaigns, such as those for parental mediation and awareness of SHS, can be implemented. The results of this study indicate directions for initiating, implementing, and evaluating programs and services for HTP use and SHS exposure in Korean adolescents. HTP use and SHS exposure may help explain risk behaviors that may motivate suicide-related perceptions and behaviors.

## Data Availability

The data supporting this study are available at the following link http://www.kdca.go.kr/yhs/home.jsp. Data were obtained from the 2019 KYRBWS, which has been conducted annually by the Korea Centers for Disease Control and Prevention (KCDC).

## References

[1] Centers for Disease Control and Prevention Adolescent health.

[2] Organization for Economic Co-Operation and Development Suicide rates.

[3] Ministry of Education, Ministry of Health and Welfare, Korea Centers for Disease Control and Prevention The 15th Korea Youth Risk Behavior Web-Based Survey.

[4] Nock MK, Green JG, Hwang I (2013). Prevalence, correlates, and treatment of lifetime suicidal behavior among adolescents: results from the National Comorbidity Survey Replication Adolescent Supplement. JAMA Psychiatry.

[5] Lange S, Koyanagi A, Rehm J, Roerecke M, Carvalho AF (2020). Association of tobacco use and exposure to SHS with suicide attempts among adolescents: findings from 33 countries. Nicotine Tob Res.

[6] Poorolajal J, Darvishi N (2016). Smoking and Suicide: A Meta-Analysis. PLoS One.

[7] Bohnert KM, Ilgen MA, McCarthy JF, Ignacio RV, Blow FC, Katz IR (2014). Tobacco use disorder and the risk of suicide mortality. Addiction.

[8] Lee Y, Lee KS (2019). Association of Depression and Suicidality with Electronic and Conventional Cigarette Use in South Korean Adolescents. Subst Use Misuse.

[9] Ministry of Education, Ministry of Health and Welfare, Korea Centers for Disease Control and Prevention The 14th Korea Youth Risk Behavior Web-Based Survey.

[10] Kim J, Yu H, Lee S, Paek YJ (2018). Awareness, experience and prevalence of heated tobacco product, IQOS, among young Korean adults. Tob Control.

[11] Grana RA, Ling PM, Benowitz N, Glantz S (2014). Electronic cigarettes. Circulation.

[12] World Health Organization Heat-not-Burn tobacco products information sheet.

[13] Korean Ministry of Economy and Finance Tobacco Market Trends.

[14] Kang SY, Lee S, Cho HJ (2021). Prevalence and predictors of heated tobacco product use and its relationship with attempts to quit cigarette smoking among Korean adolescents. Tob Control.

[15] Park S, Romer D (2007). Associations between smoking and depression in adolescence: an integrative review. Taehan Kanho Hakhoe Chi.

[16] Paperwalla KN, Levin TT, Weiner J, Saravay SM (2004). Smoking and depression. Med Clin N Am.

[17] Anderson AM, Happ MB (2021). The three-step theory of suicide: analysis and evaluation. ANS Adv Nurs Sci.

[18] Kyron MJ, Carrington-Jones P, Page AC, Bartlett J, Lawrence D (2020). Factors differentiating adolescents who consider suicide and those who attempt: Results from a National Survey of Australian Adolescents. Aust J Psychol.

[19] Imperato PJ (1983). The administration of a public health agency. A case study of the New York City Department of Health.

[20] Chen VCH, Kuo CJ, Wang TN (2015). Suicide and other-cause mortality after early exposure to smoking and secondhand smoking: a 12-year population-based follow-up study. Plos One.

[21] Baiden P, LaBrenz CA, Asiedua-Baiden G, Muehlenkamp JJ (2020). Examining the intersection of race/ethnicity and sexual orientation on suicidal ideation and suicide attempt among adolescents: findings from the 2017 Youth Risk Behavior Survey. J Psychiatr Res.

[22] Jang BN, Jeong W, Kang SH, Jang SI (2020). Association between the location of SHS exposure and depressive symptoms among South Korean adolescents. Int J Environ Res Public Health.

[23] Im Y, Oh WO, Suk M (2017). Risk Factors for Suicide Ideation Among Adolescents: Five-Year National Data Analysis. Arch Psychiatr Nurs.

[24] Hughes JR (2008). Smoking and suicide: a brief overview. Drug Alcohol Depend.

[25] Auer R, Concha-Lozano N, Jacot-Sadowski I, Cornuz J, Berthet A (2017). Heat-not-burn tobacco cigarettes: smoke by any other name. JAMA Intern Med.

[26] Obertova N, Navratil T, Zak I, Zakharov S (2020). Acute exposures to e-cigarettes and heat-not-burn products reported to the Czech Toxicological Information Centre over a 7-year period (2012-2018). Basic Clin Pharmacol Toxicol.

[27] Hamer M, Ford T, Stamatakis E, Dockray S, Batty GD (2011). Objectively measured secondhand smoke exposure and mental health in children: evidence from the Scottish Health Survey. Arch Pediatr Adolesc Med.

[28] Kim JS, Kim K (2021). Electronic cigarette use and suicidal behaviors among adolescents. J Public Health (Oxf).

[29] Kalkhoran S, Glantz SA (2016). E-cigarettes and smoking cessation in real-world and clinical settings: a systematic review and meta-analysis. Lancet Respir Med.

[30] Li D, Yang X, Ge Z (2012). Cigarette smoking and risk of completed suicide: a meta-analysis of prospective cohort studies. J Psychiatr Res.

[31] O’Brien RM (2007). A caution regarding rules of thumb for variance inflation factors. Qual Quant.

[32] Kim SH, Cho HJ (2020). Prevalence and correlates of current use of heated tobacco products among a nationally representative sample of Korean adults: Results from a cross-sectional study. Tob Induc Dis.

[33] Grana R, Benowitz N, Glantz SA (2014). E-cigarettes: a scientific review. Circulation.

[34] Fergusson DM, Woodward LJ, Horwood LJ (2000). Risk factors and life processes associated with the onset of suicidal behaviour during adolescence and early adulthood. Psychol Med.

[35] Spear LP (2000). The adolescent brain and age-related behavioral manifestations. Neurosci Biobehav Rev.

[36] Lechner WV, Janssen T, Kahler CW, Audrain-McGovern J, Leventhal AM (2017). Bi-directional associations of electronic and combustible cigarette use onset patterns with depressive symptoms in adolescents. Prev Med.

[37] DeBry SC, Tiffany ST (2008). Tobacco-induced neurotoxicity of adolescent cognitive development (TINACD): a proposed model for the development of impulsivity in nicotine dependence. Nicotine Tob Res.

[38] Malone KM, Waternaux C, Haas GL, Cooper TB, Li S, Mann JJ (2003). Cigarette smoking, suicidal behavior, and serotonin function in major psychiatric disorders. Am J Psychiatry.

[39] U.S. Food and Drug Administration 2018 NYTS Data: A Startling Rise in Youth E-Cigarette Use.

[40] Bağrul D, Arıkan Fİ (2020). The relationship between suicide attempt and smoking, alcohol and substance use and psychosocial characteristics in adolescents. Cumhuriyet Tıp Dergisi.

