# Corrigendum: Association of heated tobacco product use and secondhand smoke exposure with suicidal ideation, suicide plans, and suicide attempts among Korean adolescents: A 2019 national survey

**DOI:** 10.18332/tid/144085

**Published:** 2022-01-21

**Authors:** Soyoon Park, Kang-Sook Lee

**Affiliations:** 1 Department of Public Health, Graduate School, The Catholic University of Korea, Seoul, Korea; 2 Department of Preventive Medicine, College of Medicine, The Catholic University of Korea, Seoul, Korea

**Keywords:** heated tobacco product, SHS, smoking, suicide, adolescents

Corrigendum on:

Association of heated tobacco product use and secondhand smoke exposure with suicidal ideation, suicide plans, and suicide attempts among Korean adolescents: A 2019 national survey By Soyoon Park, Kang-Sook Lee Tobacco Induced Diseases, Volume 19, Issue September, Pages 1–11, Publish date: 21 September 2021 DOI: https://doi.org/10.18332/tid/140824

The manuscript type is ‘Research Paper’ instead of ‘Review Paper’.

